# *Curled Flag Leaf 2*, Encoding a Cytochrome P450 Protein, Regulated by the Transcription Factor *Roc5*, Influences Flag Leaf Development in Rice

**DOI:** 10.3389/fpls.2020.616977

**Published:** 2021-02-12

**Authors:** Xiaobo Zhang, Ying Wang, Xiaoyan Zhu, Xiaowen Wang, Zhu Zhu, Yangyang Li, Jia Xie, Yuzhen Xiong, Zhenglin Yang, Guanghua He, Xianchun Sang

**Affiliations:** Key Laboratory of Application and Safety Control of Genetically Modified Crops, Rice Research Institute, Southwest University, Academy of Agricultural Sciences, Chongqing, China

**Keywords:** cell wall, *CFL2/OsCYP96B4*, curled flag leaf, epidermis, L1 box, rice, *Roc5*

## Abstract

Moderate curling generally causes upright leaf blades, which favors the establishment of ideal plant architecture and increases the photosynthetic efficiency of the population, both of which are desirable traits for super hybrid rice (*Oryza sativa* L.). In this study, we identified a novel curled-leaf mutant, *curled flag leaf 2* (*cfl2*), which shows specific curling at the base of the flag leaf owing to abnormal epidermal development, caused by enlarged bulliform cells and increased number of papillae with the disordered distribution. Map-based cloning reveals that *CFL2* encodes a cytochrome P450 protein and corresponds to the previously reported OsCYP96B4. *CFL2* was expressed in all analyzed tissues with differential abundance and was downregulated in the *clf1* mutant [a mutant harbors a mutation in the homeodomain leucine zipper IV (HD-ZIP IV) transcription factor *Roc5*]. Yeast one-hybrid and transient expression assays confirm that *Roc5* could directly bind to the *cis*-element L1 box in the promoter of *CFL2* before activating *CFL2* expression. RNA sequencing reveals that genes associated with cellulose biosynthesis and cell wall-related processes were significantly upregulated in the *cfl2* mutant. The components of cell wall, such as lignin, cellulose, and some kinds of monosaccharide, were altered dramatically in the *cfl2* mutant when compared with wild-type “Jinhui10” (WT). Taken together, *CFL2*, as a target gene of *Roc5*, plays an important role in the regulation of flag leaf shape by influencing epidermis and cell wall development.

## Introduction

The leaf is the primary photosynthesis organ in plants. The morphological characteristics of leaves, such as shape, size, and thickness, directly affect light utilization and thus influence the yield of crops (Zhang et al., 2015). Moderate curling is beneficial for the development of an erect leaf, which improves the population structure and increases the light utilization, and has important outcomes for breeding high-yield rice (Richards et al., 2002; Wang et al., 2020). Therefore, elucidation of the genetic mechanism of leaf curling is important both in understanding leaf development and in the improvement of plant architecture in rice.

Leaf curling is a complex agronomic character, which is regulated by both genotype and environment (Li et al., 2017). The rolling rice leaf is affected by bulliform cell development, adaxial–abaxial axis polarity, sclerenchyma formation, and epidermal structure (Xu et al., 2013). The morphology, number, size, and distribution of the bulliform cells in the epidermis are the predominant factors that affect leaf rolling. The transferred DNA (T-DNA) mutant *BY240*, in which the T-DNA was inserted in the promoter of *ABAXIALLY CURLED LEAF1* (*ACL1*), shows increased number, and size of bulliform cells, which causes uncoordinated development of the abaxial and adaxial epidermis of the leaf, and results in abaxially rolled leaves. Overexpression of *ACL2*, the paralog of *ACL1*, can also induce abaxial leaf rolling (Li et al., 2010). Many additional genes are involved in leaf rolling by affecting the bulliform cell development, such as *SEMI-ROLLED LEAF1* (*SRL1/CLD1*) (Xiang et al., 2012; Li et al., 2017), *ZINC-FINGER HOMEODOMAIN1* (*OsZHD1*) (Xu et al., 2014), *RICE OUTERMOST CELL-SPECIFIC GENE5* (*Roc5*) (Zou et al., 2011), *ROLLED AND ERECT LEAF1* (*REL1*) (Chen et al., 2015), *REL2* (Yang et al., 2016), *LATERAL ORGAN BOUNDARIES DOMAIN* (*OsLBD3-7*) (Li et al., 2016), and *SHALLOT-LIKE2* (*SLL2*) (Zhang et al., 2015), but the mechanisms remain unclear. In addition, genes influence the bulliform cells through regulation of phytohormone, such as *YABBY1* (*OsYAB1*) in gibberellin biosynthesis (Dai et al., 2007), *BRASSINOSTEROID INSENSITIVE1-ASSOCIATED KINASE1* (*OsI-BAK1*) in brassinosteroid (BR) signaling (Khew et al., 2015), and *CONSTITUTIVELY WILTED1* (*OsCOW1*/*NAL7*) and *AUXIN RESPONSE FACTOR18* (*OsARF18*) in indole-3-acetic acid (IAA) synthetic pathway (Woo et al., 2007; Fujino et al., 2008; Huang et al., 2016). And, some genes related with secondary cell wall or cellulose formation, such as *CELLULOSE SYNTHASE-LIKE D4* (*OsCSLD4/NRL1*) (Li et al., 2009; Hu et al., 2010; Luan et al., 2011), *ROLLING LEAF14* (*RL14*) (Fang et al., 2012), and *OsMYB103L* (Yang et al., 2014), also influence the bulliform cell development.

Curling of the leaf usually correlates with polarity changes in the abaxial–adaxial axis. Homeodomain leucine zipper class III (HD-ZIPIII) family members are involved in the establishment of polarity during leaf development (McConnell et al., 2001; Otsuga et al., 2001; Juarez et al., 2004; Nagasaki et al., 2007). Five HD-ZIP III genes have been identified in rice. *OSHB3* contributes to leaf polarity more than *OSHB1* by involvement in vascular patterning and differentiation, whereas transgenic plant harboring mutated *OSHB5* does not exhibit defective leaf polarity (Itoh et al., 2008). *ADXIALIZED LEAF1* (*ADL1*) encodes a calpain-like cysteine proteinase, and loss-of-function of the maize ortholog *DEFECTIVE KERNEL1* (*DEK1*) alters the adaxial–abaxial axis of the leaves, which shows ectopic bulliform-like cells in the abaxial epidermis (Hibara et al., 2009). *OsAGO7* is a direct homolog of the *Arabidopsis thaliana ZIP/Ago7* gene, which is involved in polar patterning, and controls the upward curling of rice leaves (Shi et al., 2007).

Sclerenchyma cells are lignified dead cells with thickened secondary cell walls that surround the vascular bundle and are involved in leaf rolling in rice (Zhang et al., 2009). *SLL1* encodes a MYB transcription factor belonging to the KANADI family, and *SRL2* encodes a novel plant-specific protein with unknown function. Mutants of both genes develop rolled leaves caused by the abnormal development of sclerenchyma cells on the abaxial side of the leaf (Zhang et al., 2009; Liu et al., 2016). Analysis of the *srl2 sll1* double mutant shows that *SLL1* and *SRL2* participate in distinct metabolic pathways to regulate the development of sclerenchyma cells in the rice leaf (Zhang et al., 2009).

Epidermal structure also affects the leaf rolling. *CURLY FLAG LEAF1* (*CFL1*), which encodes a protein containing a WW domain, interacts with *HOMEODOMAIN GLABROUS1* (*HDG1*) to regulate negatively cuticle development, and the impaired cuticle formation is responsible for the curly leaf in *cfl1* mutant (Wu et al., 2011). Obvious defects in the leaf epidermis and cuticle structures in the *cld1* mutant, such as the disordered distribution of papillae and linear cork–silica cell pairs, indicate the importance of epidermal integrity for the maintenance of leaf shape (Li et al., 2017).

Although several of genes associated with leaf rolling have been identified and cloned, the mechanisms of abnormal leaf morphology are still poorly understood. In this study, we identified a novel curled-leaf mutant *cfl2*, generated by ethyl methane sulfonate (EMS) mutagenesis, which showed specific curling at the base of the flag leaf. Map-based cloning revealed that *CFL2* encodes the OsCYP96B4 protein, which is a member of the cytochrome P450 monooxygenase family. Histological analysis and observation using scanning electron microscopy (SEM) revealed abnormal epidermis development in the *cfl2* mutant. Biochemical and transcriptome analyses suggest that *CFL2* is a downstream target gene of the HD-ZIP IV transcription factor *Roc5*, and upregulated genes are involved in cell wall-related processes in *cfl2* mutant, which led to the abnormal development of the epidermis and thereby modulate flag leaf shape.

## Materials and Methods

### Plant Materials and Growth Conditions

The rice curled flag leaf mutant *cfl2* and its two allelic mutants *cfl2-1 and cfl2-2* were isolated from the progeny of *indica* restorer line “Jinhui10” seeds treated with EMS. The F_2_ mapping population was raised from the cross between the *cfl2* and “Xinong1A,” a sterile line, with normal plant height and leaf morphology, selected by the Rice Research Institute of Southwest University. All plants were grown in paddy fields under natural conditions at the Rice Research Institute of Southwest University in Chongqing, China.

### Microscopic Observations

For paraffin sectioning, the base of flag leaf of the wild-type “Jinhui10” (WT) and the *cfl2* mutant were fixed with FAA solution (45% water, 45% ethanol, 5% formaldehyde, and 5% acetic acid) for 2 days at 4°C. The samples were embedded in paraffin after dehydrating with a graded ethanol series and infiltrating with ethanol–xylene in different proportions. Thin sections (approximately 8 μm thick) were cut using a rotary microtome (RM2245, Leica Microsystems, Hamburg, Germany). The sections were stained with fast green and safranin, then observed and photographed using a light microscope (Eclipse Ci-L, Nikon, Tokyo, Japan), as described previously (Ma et al., 2017). For frozen sections, the fresh flag leaf base was embedded in NEG-50 Frozen Section Medium and cut into 8 μm-thick sections at −20°C using a cryostat microtome (CryoStar NX50 OP, Thermo Fisher Scientific, United States), then observed and photographed through a light microscope (Eclipse Ci-L, Nikon, Tokyo, Japan). The area of bulliform cells was measured with AxioVision release 4.6 software. For SEM observations, fresh leaf samples were prepared and examined using a Hitachi SU3500 scanning electron microscope (Zhang et al., 2017).

### Map-Based Cloning

A total of 837 individuals with curled flag leaves, which were selected from the 2,582 individuals of the F_2_ population raising from the cross between *cfl2* mutant and “Xinong1A,” were used for *CFL2* mapping. Simple sequence repeat (SSR) markers obtained from the publicly available rice databases^1^ were utilized for initial mapping, and insertion/deletion markers developed by our laboratory were used for fine mapping. For functional complementation, the *CFL2* genomic fragment, consisting of 2,877 bp upstream of the start codon, the open reading frame, and 899 bp downstream of the stop codon, was amplified from the WT genomic DNA, digested using *Eco*RI and *Kpn*I, and inserted into the binary vector pCAMBIA1301. The *CFL2* complementation plasmid was introduced into the *cfl2* mutant by *Agrobacterium*-mediated transformation as described previously (Sang et al., 2012). The primers used in this study are listed in Supplementary Table S4.

### Subcellular Localization

The full-length coding sequence of *CFL2* without the stop codon was amplified from WT and digested using *Spe*I and *Bam*HI and then fused to the N-terminus of the *GREEN FLUORESCENT PROTEIN* (*GFP*) gene under the control of the enhanced *Cauliflower mosaic virus* (CaMV) 35S promoter in the expression vector pAN580 to generate CFL2–GFP construct. The CFL2–GFP plasmids were transformed into rice protoplasts by polyethylene glycol-mediated method. After overnight incubation at 28°C, fluorescence was observed using a LSM800 confocal laser microscope (Zeiss, Jena, Germany).

### Protein Sequence Alignment and Phylogenetic Analysis

The full-length amino acid sequences of CYP96 clan members in rice and *Arabidopsis* were downloaded from the National Center for Biotechnology Information (NCBI) databases^2^. The protein sequence of ATHDG1 was downloaded from TAIR^3^, and the homologous genes in rice were identified from Phytozome^4^. Multiple sequence alignment was generated using ClustalX2.1. Phylogenetic trees were reconstructed with MEGA 5 using the maximum likelihood method and bootstrapping (with 1,000 replicates).

### *In situ* Hybridization

The 387-bp *CFL2* probe was amplified from the cDNA and labeled using the DIG RNA Labeling Kit (Roche, Basel, Switzerland) in accordance with the manufacturer’s protocols. The leaf and shoot apical meristem (SAM) of the WT were fixed in FAA overnight at 4°C. Pretreatment of sections, hybridization, and immunological detection were performed following previously described methods (Sang et al., 2012).

### RNA Isolation and Quantitative Real-Time PCR Analysis

Total RNA of various plant tissues of the WT and mutants were isolated using the RNAprep Pure Plant Kit (Tiangen, Beijing, China). The concentration, purity, and integrity of extracted RNA were determined using a NanoDrop One spectrophotometer (Thermo Fisher Scientific) and agarose gel electrophoresis. RNA reverse transcription was carried out using the SuperScript III Reverse Transcriptase Kit (Invitrogen, Carlsbad, CA, United States) in accordance with the manufacturer’s instructions. Quantitative real-time PCR (qRT-PCR) was performed using NovoStart SYBR qPCR SuperMix Plus (Novoprotein, Shanghai, China) and a CFX Connect^TM^ Real-time System (Bio-Rad, Hercules, CA, United States) with three replicates.

### Yeast One-Hybrid Assay

For the yeast one-hybrid assay (Y1H), the three tandems repeated L1 box AACATTTA and L1 mutant box AACCGTTA oligonucleotide sequences were annealed and inserted into the *Sac*I and *Sal*I sites in the pAbAi plasmid to generate pL1–AbAi and pL1mutant–AbAi. The two plasmids, linearized with *Bst*BI, were transformed into Y1HGold cells on SD/-URA agar medium using the protocol for the Yeastmaker Yeast Transformation System to generate Y1HGold (L1/AbAi) and Y1HGold (L1 mutant/AbAi) strains. The full-length coding sequence (CDS) of *Roc5* was amplified and cloned into the pGADT7 vector to generate *Roc5–*AD plasmids and then transformed to the two newly constructed Y1HGold bait strains after testing their background for AbA^*r*^ expression.

### Dual-Luciferase Assay

For the dual-luciferase assays, a 2.1 kb promoter segment of *CFL2* was cloned and recombined into pGreenII 0800-LUC vector digested with *Kpn*I and *Hin*dIII, thus generating the p*CFL2*:LUC reporter construct. The Renilla luciferase gene driven by CaMV35S promoter in the pGreenII 0800-LUC vector was used as an internal reference. The full-length CDS of *Roc5* was amplified and inserted into the *Bam*HI and *Eco*RI sites of the pGreenII 62-SK vector driven by the 35S promoter as the effector. The empty pGreenII 62-SK vector was used as a control. The dual-luciferase assays were performed in rice protoplasts, and the luciferase signal was detected using Dual-Luciferase Reporter Assay System and GloMax^®^ 20/20 Luminometer (Promega, Madison, WI, United States) following the manufacturer’s instructions.

### Transcriptome and Gene Ontology Enrichment Analyses

Total RNA was isolated from the base of the flag leaf of WT and *cfl2* with three biological replicates at the early stage before the flag leaf emerged from the sheath. Library preparation and sequencing of six libraries were conducted by the Biomarker Technologies Corporation (Beijing, China) using a HiSeq 4000 platform (Illumina, San Diego, CA, United States) following the manufacturer’s protocol. The raw reads were filtered and then mapped to the rice reference genome using HISAT2 software with default parameters (Kim et al., 2015). Gene expression levels were quantified using StringTie and expressed as fragments per kilobase of transcript per million mapped reads (FPKM) (Florea et al., 2013). The differentially expressed genes (DEGs) were detected using the DESeq R package with the following criteria: false discovery rate < 0.05 and log_2_| fold change| > 1 (Love et al., 2014). The DEGs were used for gene ontology (GO) enrichment analysis with the agriGO online resource^5^. RNA sequencing data were deposited in the NCBI Sequence Read Archive (SRA) under accession PRJNA628038.

### Analysis of Cell Wall Components

The base of flag leaves was collected from WT and *cfl2* mutant for the cell wall component analysis. The lignin and cellulose content was measured using the correspondent kit (Solarbio, Catalog no. BC4205 and BC4285, respectively). The monosaccharide was extracted and analyzed by high-performance liquid chromatography (HPLC). To the above 0.1 g sample, 0.5 ml precooled trifluoroacetic acid (4 mol/l) was added and hydrolyzed for 4 h under 110°C, and the supernatant was collected after 8,000 *g* centrifugation for 10 min. Then, methanol (500 μl) was added, blow dried by nitrogen, and dissolved into 500 μl water. Solving liquid (50 μl) was taken and NaOH (50 μl) and PMP methanol solution (100 μl) were added and reacted for 100 min at 70°C avoiding light. HCl (100 μl) and water (400 μl) were added to the reaction mixture and then extracted by the same volume of chloroform three times. The 10 μl extractant was analyzed by HPLC (RIGOL L3000) with a column (RIGOL C18, 250 mm × 4.6 mm, 5 μm) at 30°C.

## Results

### Phenotypes of the *cfl2* Mutant

**FIGURE 1:**
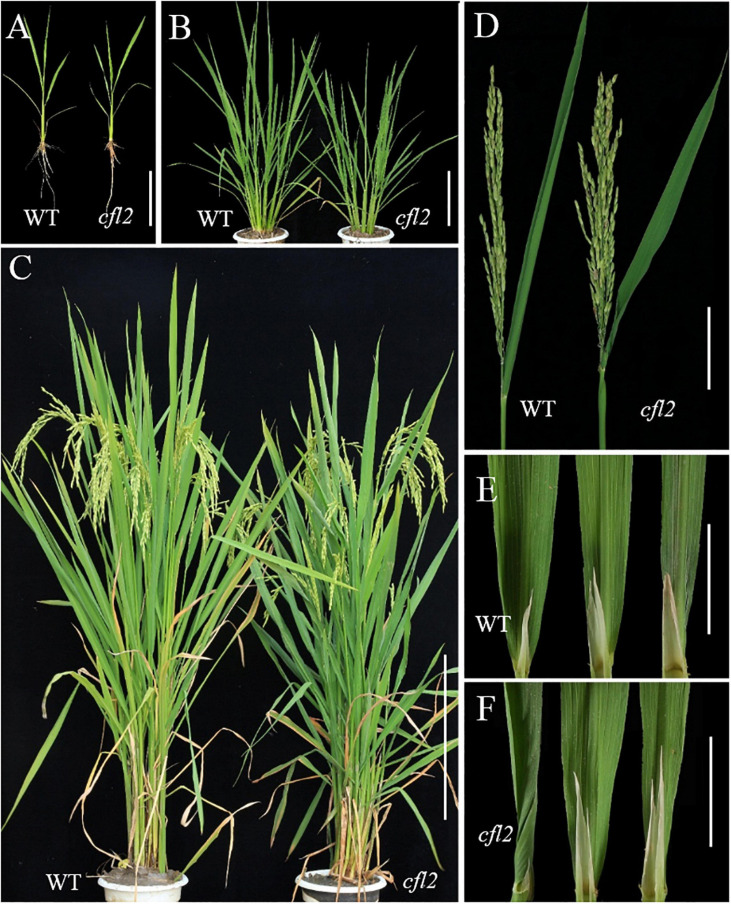
Phenotypic identification of the WT and *cfl2* mutant. **(A)** 2 week-old seedlings of WT and *cfl2*. Scale bar = 12 cm. **(B)** WT and *cfl2* plants in tillering stage. Scal bar = 20 cm. **(C)** Mature WT and *cfl2* plants. Scale bar = 30 cm. **(D)** Panicle and flag leaf of WT and *cfl2.* Scale bar = 10 cm. **(E,F)** The top three leaves of WT and *cfl2*. Scale bars = 3 cm.

The *cfl2* mutant was identified from the progeny of EMS-treated seeds of the rice *indica* restorer line “Jinhui10.” In the paddy field, the *cfl2* mutant was slightly and insignificantly shorter than WT at seedling stage, but the leaf morphology of the *cfl2* mutant was identical to that of the WT, as well as the root and tiller (Figures 1A,B). At heading stage, no significant difference in plant height was observed between WT and the *cfl2* mutant (Figure 1C and Supplementary Figure S1). The main agronomic traits of the *cfl2* mutant were normal compared with those of WT (Supplementary Table S1). The flag leaf base of the *cfl2* mutant was abnormal, in that the basal half of the leaf was curled inward, whereas the middle and distal portions were normally developed (Figure 1D). In addition, the blade of other leaves of the *cfl2* mutant, including the second and third upper leaves, were normally unfolded as in the WT (Figures 1E,F). Two allelic mutants *cfl2-1* and *cfl2-2* were further identified from the mutant library. Although both of the allelic mutants exhibited a dwarfism phenotype, which is different from the *cfl2*, their flag leaves were also specifically curly (Supplementary Figure S2). These results indicate that the *cfl2* was a specific curling mutant at the base of the flag leaf.

### Flag Leaf Microstructure of the *cfl2* Mutant

**FIGURE 2:**
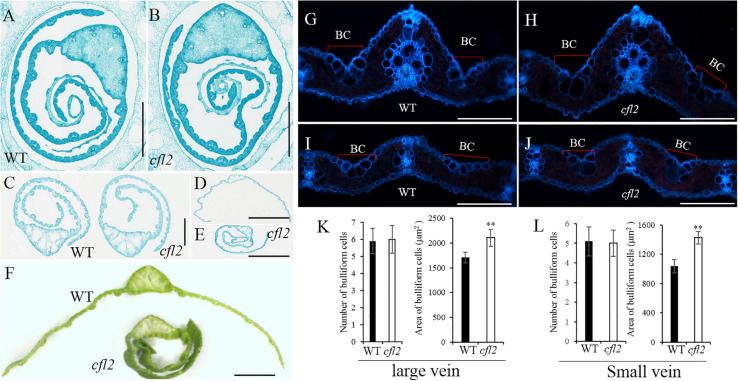
Flag leaf microstructure of WT and *cfl2*. **(A,B)** The WT and *cfl2* flag leaf wrapped by sheath. Scale bars = 3 mm. **(C)** The emergence flag leaf of WT and *cfl2*. Scale bars = 1 mm. **(D–F)** The fully expanded flag leaf of WT and *cfl2*. Scale bar = 3 mm **(D,E)** and 1 mm **(F)**. **(G–J)** Frozen cross sections of large vascular and small vascular bundles of WT and *cfl2*. BC, bulliform cells. Scale bars = 100 μm. **(K,L)** Statistical analysis of number and area of bulliform cells of large vascular and small vascular bundles in WT and *cfl2*. Values are means ± SD (*n* = 10). Student’s *t*-test was used for statistical analysis (^∗∗^*P* < 0.01).

To further characterize the morphology of the flag leaf base, paraffin-embedded sections of the flag blade base at different stages were prepared. Both sides of the WT flag leaf blade were curled in a regular, circular manner, whereas the inner side of the *cfl2* mutant was arranged irregularly at the early stage of blade development (Figures 2A,B). This irregular curling phenotype in the mutant continued after the flag leaf emergence (Figure 2C). When the flag leaf was fully expanded, both sides of the leaf blade showed outward expansion in the WT. In the *cfl2* mutant, the outside half of the leaf blade showed outward expansion, whereas the inside half of the blade showed adaxial curling (Figures 2D,E), which ultimately formed a shallot-like structure (Figure 2F). Frozen cross sections of the mature flag leaf revealed that the number of bulliform cells on the sides of large and small vascular bundles was unchanged between the WT and *cfl2* mutant. However, the average area of bulliform cells was significantly larger in the *cfl2* mutant than in the WT (Figures 2G–L and Supplementary Table S5), which suggests that the curled leaf in the *cfl2* mutant may be caused by the enlarged bulliform cells.

**FIGURE 3:**
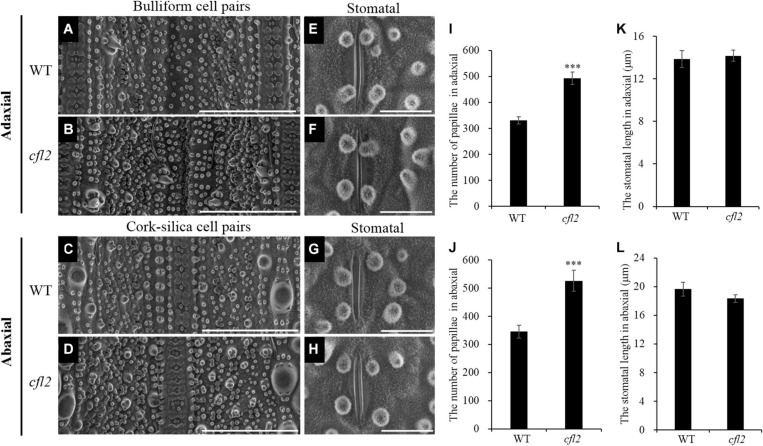
SEM observations of the flag leaf epidermis in WT and *cfl2*. **(A,B)** The bulliform cell pairs of WT and *cfl2* in adaxial epidermis. Scale bars = 100 μm. **(C,D)** The cork–silica cell pair area of WT and *cfl2* in abaxial epidermis. Scale bars = 100 μm. **(E–H)** The stomata of WT and *cfl2* in adaxial and abaxial axis. Scale bars = 10 μm. **(I,J)** Statistical analysis of the number of papillae cell in adaxial and abaxial axis of WT and *cfl2*. **(K,L)** Statistical analysis of the stomatal length in adaxial and abaxial axis of WT and *cfl2*. Values are means ± SD (*n* = 6). Student’s *t*-test was used for statistical analysis (^∗∗∗^*P* < 0.001).

Marked differences between the WT and *cfl2* mutant in the epidermis structure of the flag leaf were observed by SEM. The epidermal of bulliform cell pairs was smooth in the WT, but a distinct crack was observed in the *cfl2* mutant (Figures 3A,B). The papillae were sparse and regularly arranged in both of the adaxial and abaxial epidermis of WT flag leaf, whereas the number of papillae was increased highly significant and their distribution disordered in the flag leaf of the *cfl2* mutant (Figures 3A–D,I,J). No significant difference was observed between the WT and *cfl2* mutant in the stomata and the crystal structure of cuticular wax on the adaxial and abaxial surfaces of the flag leaf (Figures 3E–H,K,L). These results demonstrated that the epidermal modifications were responsible for the curled flag leaf in the *cfl2* mutant.

### Map-Based Cloning of *CFL2*

**FIGURE 4:**
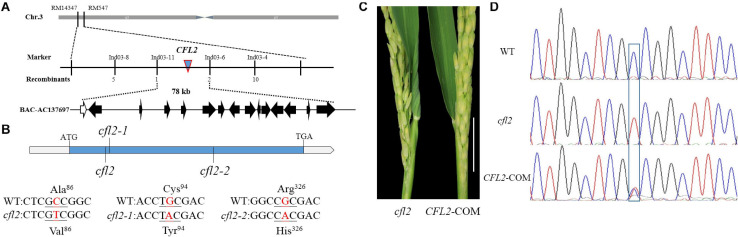
Map-based cloning of *CFL2*. **(A)** The *CFL2* gene was fine mapped to the long arm of chromosome 3 between Ind03-11 and Ind03-6 with a physical interval 78 kb. **(B)** Gene structure of *CFL2* and the mutation sites in *cfl2* and its allelic mutant *cfl2-1* and *cfl2-2.* **(C)** The base of the flag leaf morphology of the *cfl2* mutant and complemented transgenic plant (*CFL2*-COM). Scale bar = 5 cm. **(D)** Sequence peak chromatograms of the mutation region in plants of the WT, *cfl2*, and *CFL2*-COM.

To clone *CFL2*, the *cfl2* mutant was crossed with “Xinong 1A,” an *indica* male-sterile line. The plant phenotypes, including the flag leaf morphology, were identical to those of the WT in all F_1_ individuals. Genetic analysis showed that segregation of the mutant phenotype in the F_2_ progeny conformed to a 3:1 ratio (837 of 2,582 individuals showed the mutant phenotype; *χ*^2^ = 0.49 < *χ*^2^_0_._05_ = 3.84), which indicated that the curled flag leaf trait of the *cfl2* mutant was controlled by a single recessive nuclear gene. A total of 837 individuals consistent with the mutant phenotype were used for mapping analysis. *CFL2* was fine mapped to the long arm of the chromosome 3 between InDel marker Ind03-11 and Ind03-6 with a physical interval of 78 kb (Figure 4A). Sequencing the 15 genes annotated in the Gramene database^6^ within the interval (Supplementary Table S2) revealed a single-nucleotide transition (C to T, causing a substitution of Ala to Val) within *LOC_Os03g04680* in the *cfl2* mutant. The two allelic mutants *cfl2-1* and *cfl2-2* were showed to be mutated in the coding sequence, leading to the substitution of the amino acid at different locations (Figure 4B).

To verify that *LOC_Os03g04680* is equivalent to the *CFL2* gene, genome complementation was performed. The flag leaf base of the complemented line was recovered (Figure 4C), and the complemented lines were heterozygous (C/T) at the substitution site (Figure 4D). These results confirmed that the mutation of *LOC_Os03g04680* was responsible for the curled flag leaf phenotype of the *cfl2* mutant.

### *CFL2* Encodes a Cytochrome P450 Protein

**FIGURE 5:**
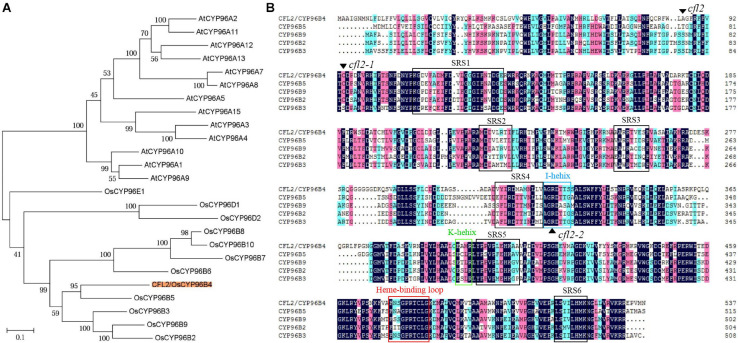
Protein bioinformatics analysis of CFL2. **(A)** Phylogenetic tree of CYP96 subfamily in rice and *Arabidopsis*. **(B)** Multiple sequence alignment of the CYP96 subfamily gene cluster on chromosome 3. The *black arrowhead* indicates the amino acid residue mutated in mutants. The *open box with different color* represent the conserved structures domains of CYP proteins, including SRS (substrate-recognition sites), I-helix (I-helix groove), K-helix (Glu-x-x-Arg domain), and a heme binding loop.

*LOC_Os03g04680* encodes a cytochrome P450 protein, belonging to the CYP96 subfamily, which was previously designated *OsCYP96B4* (Nelson et al., 2004; Rengasamy et al., 2011). In total, 12 and 13 CYP96 members have been identified in the *Arabidopsis* and rice genome, respectively. Phylogenetic analysis showed that 25 proteins were categorized into two distinct evolutionary branches, which indicated that CYP96 subfamily proteins show strong functional conservations in monocotyledons and dicotyledons (Figure 5A). The CYP96 proteins contain a number of highly conserved domains, including six substrate-recognition sites (SRS), an I-helix, a K-helix (Glu-x-x-Arg domain), and a heme binding loop (Schuler and Werck-Reichhart, 2003). The amino acid sequence alignment showed that the *cfl2* mutation site was not within a conserved domain. However, the mutation of the two allelic mutants *cfl2-1* and *cfl2-2* located the different conserved domain, especially the *cfl2-2* mutation site, which was located in the I-helix domain (Figure 5B). The different mutation sites may be the cause of the different phenotypes in plant height of the three *CFL2* allelic mutants.

### Expression Pattern of *CFL2*

**FIGURE 6:**
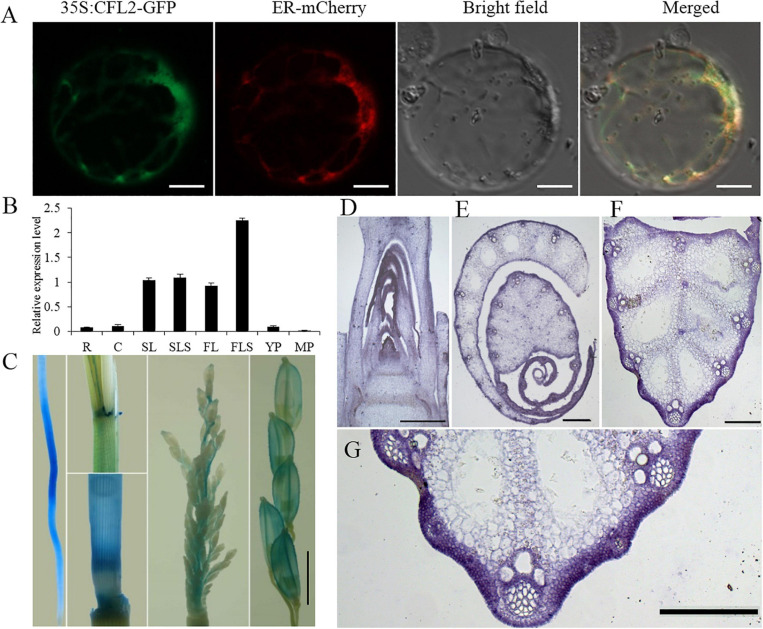
Protein subcellular localization and expression pattern of *CFL2*. **(A)** Subcellular localization of *CFL2–*GFP fusion protein in rice protoplasts. Scale bars = 10 μm. **(B)** qRT-PCR analysis of *CFL2* in various organs, including the root (R), culm (C), second leaf (SL), second leaf sheath (SLS), flag leaf (FL), flag leaf sheath (FLS), young panicle (YP), and mature panicle (MP). Values are means ± SD (*n* = 3). **(C)** GUS stains in root, leaf sheath, culm, young panicle, and mature panicle. Scale bar = 2 mm. **(D–H)** *In situ* hybridization analysis of *CFL2* expression in SAM **(D)**, leaf **(E)**, leaf vein **(F)**, its partial enlarged drawing **(G)**, and the control tissue using a sense probe **(H)**. Scale bars = 1 cm **(D–G)** and 2 cm **(H)**.

To determine the subcellular localization of CFL2, the open reading frame of *CFL2* was fused to GFP and driven by the CaMV35S promoter, and the plasmid was transformed into rice protoplast. The CFL2 fusion protein was co-localized with an endoplasmic reticulum (ER) marker, which suggests that CFL2 is located on the ER membrane (Figure 6A).

To investigate the expression pattern of *CFL2*, different tissues were analyzed by qRT-PCR. *CFL2* was expressed in diverse tissues, including the root, culm, leaf and leaf sheath, youth panicle, and mature panicle. The highest level of expression was observed in the leaf blade and sheath, especially in flag leaf sheath (Figure 6B). GUS activity was ubiquitously in the root, culm sheath, and panicle of transgenic lines, which is consistent with the qRT-PCR results (Figure 6C). *In situ* RNA hybridization revealed that the *CFL2* signal was detected in the outmost cells of SAM, leaf, and leaf vein (Figures 6D–H), which shows that *CFL2* is an outermost cell layer-specific gene.

### *CFL2* May Be the Target Gene of Roc5

**FIGURE 7:**
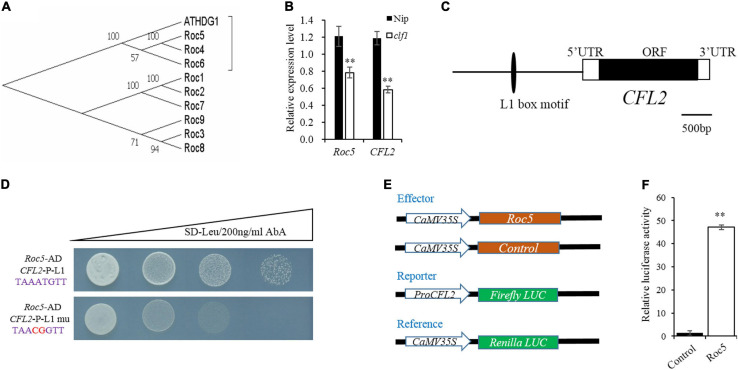
*CFL2* may be a target gene of *Roc5*. **(A)** Phylogenetic tree of ATHDG1 and the nine numbers of HD-ZIP IV family gene in rice. **(B)** Expression of *Roc5* and *CFL2* in Nipponbare (Nip) and the *clf1* mutant. Values are means ± SD (*n* = 3). Student’s *t*-test was used for statistical analysis (^∗∗^*P* < 0.01). **(C)** Distribution of the L1 box motif in the promoter regions of *CFL2*. **(D)** Yeast one-hybrid assay. **(E)** Schematic diagram show the constructs used in dual-luciferase assay. The empty pGreenII 62-SK vector was used as a control. **(F)** *Roc5* activates the expression of *CFL2* in rice protoplasts. Values are means ± SD (*n* = 3). Student’s *t*-test was used for statistical analysis (^∗∗^*P* < 0.01).

Given the defective leaf epidermis of the *cfl2* mutant, and that *CFL2* was specifically expressed in the outermost cell layer, we considered that *CFL2* may be involved in the epidermis development. *CFL1* encodes a WW-domain protein and negatively regulates epidermal cuticle development in rice. The homologous *AtCFL1* in *Arabidopsis* can interact with *HDG1*, a transcription factor belonging to the HD-ZIP IV family, which is closely associated with the epidermis (Wu et al., 2011). Nine genes in the HD-ZIP IV family have been identified in rice (Ito et al., 2003), which form three distinct clades in phylogenetic trees, and the *Roc4*, *Roc5*, and *Roc6* belong to the same clade with *ATHDG1* (Figure 7A), which indicates that the three genes may show similar functions to *ATHDG1* in the epidermis. The expression levels of *Roc5* and *CFL2* were significantly decreased in the *clf1*, an allelic mutant of *Roc5* (Xing et al., 2017), compared with those of “Nipponbare” (Figure 7B). Gene sequence analysis revealed that a conservative L1 box-binding motif TAAATGTT (*CFL2*-P-L1) is present in the promoter region of *CFL2* (Figure 7C). The Y1H assay result showed that Roc5 can bind to the *CFL2*-P-L1 box but cannot bind to the *CFL2*-P-L1 mutant box when selected with 200 ng/ml AbA in Y1H strains (Figure 7D). In addition, a dual-luciferase assay with rice protoplasts confirms that Roc5 can activate LUC transcription through binding to the *CFL2* promoter (Figures 7E,F). These results suggested that *Roc5* binds to the L1 box in the promoter of *CFL2* and regulates its expression.

### Identification and Functional Classification of DEGs Between WT and *cfl2*

**FIGURE 8:**
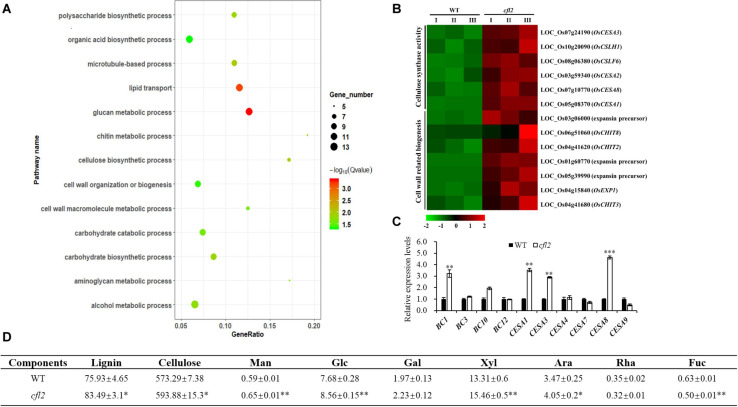
The analysis of cell wall-related process in *cfl2*. **(A)** GO enrichment of 789 upregulated genes in *cfl2* transcriptome dates. The enriched pathways are listed on the left. **(B)** Heatmap showing the expression patterns of selected genes in the process of cellulose synthase and cell wall-related process. Data are from three biological replicates (I, II, and III). **(C)** Relative expression level of cell wall-related genes in WT and *cfl2*. Values are means ± SD (*n* = 3). Student’s *t*-test was used for statistical analysis (^∗∗^*P* < 0.01; ^∗∗∗^*P* < 0.001). **(D)** Compositional analysis of cell wall components among WT and *cfl2*. Each component was calculated as milligrams per gram. *Man*, mannose; *Glc*, glucose; *Gal*, galactose; *Xyl*, xylose; *Ara*, arabinose; *Rha*, rhamnose; *Fuc*, fucose. The results were mean ± SD of five or three independent experiments. Student’s *t*-test was used for statistical analysis (^∗^*P* < 0.05; ^∗∗^*P* < 0.01).

To further explore the function of *CFL2*, the transcriptome sequencing of the flag leaf base from the *cfl2* mutant and WT was performed. A significant positive correlation among three biological replicates (Pearson’s correlation > 0.867) was observed. After filtering, a total of 132,731,874 paired-end reads were obtained, and each biological replicate was uniquely mapped to the rice reference genome (Supplementary Table S3). A total of 1,033 DEGs between the *cfl2* mutant and WT were identified, of which 789 genes were upregulated and 244 genes were downregulated. The results of GO enrichment analysis showed that genes were significantly over-represented in carbohydrate metabolic process, polysaccharide metabolic process, lipid transport, and cellulose biosynthetic and cell wall-related processes (Figure 8A). The cellulose synthase and cellulose synthase-like gene superfamily (*CESA/CSL*) is considered to be involved in cellulose and non-cellulosic matrix polysaccharide synthesis, which is an important component of plant cell walls (Wang et al., 2010). Among members of the superfamily, *OsCESA1*, *OsCESA3*, and *OsCESA8* are required for cellulose synthesis in the primary cell walls, and *CSLH1* and *CSLF6* play important roles in the polysaccharide synthesis (Vega-Sanchez et al., 2012). Additionally, cell wall-related genes, such as expansins (*OsEXP1*), expansin precursor (*LOC_Os03g06000*, *LOC_Os01g60770*, and *LOC_Os05g39990*), and chitinase family genes (*CHIT2*, *CHIT3*, and *CHIT8*), were also upregulated in the *cfl2* mutant (Figure 8B). The expression level of several cell wall-related genes was verified by qRT-PCR, which showed a similar expression pattern in *cfl2* with the transcriptome data (Figure 8C). Meanwhile, we also found that the expression of cell wall-related genes showed the same trend in *clf1* as in *cfl2*, such as *BC1*, *CESA1*, and *CESA8* were increased significantly in *clf1*, when compared to “Nipponbare” (Supplementary Figure S4B). We further analyzed the cell wall composition of WT and *cfl2* flag leaf. The main cell wall component, lignin, and cellulose were increased significantly, and the monosaccharide, such as mannose, glucose, and xylose were increased at an extremely significant level in *cfl2* (Figure 8D and Supplementary Table S6). These results indicate the importance of *CFL2* in the cell wall processes.

## Discussion

### Curled Flag Leaf Phenotype in the *cfl2* Mutant

Leaf rolling is a common mutant phenotype in rice. Normal curling of the leaf blade in the mature stage is beneficial for the spatial structure of the population and light utilization. To date, at least 31 rolled-leaf mutants have been characterized (Zhang et al., 2015), which showed the effect on all the leaves during the whole growth period and accompanied by multi-phenotypes, such as the reduced plant height (Hibara et al., 2009; Hu et al., 2010; Xu et al., 2014), the defective reproductive development (Khew et al., 2015; Huang et al., 2016; Zhao et al., 2016), and the abnormal root growth (Woo et al., 2007; Zhang et al., 2009). Few mutants in which only the flag leaves were affected have been reported. A rolled-leaf mutant was designated *cfl1* because of the curly flag leaf phenotype, but all leaves of the mutant were affected after tillering stage (Wu et al., 2011). The flag leaf mutant *sfl1* exhibits the screw flag leaf and panicle-at-bottom phenotypes, accompanied by additional changes in agronomic characters, such as the reduced plant height, flag leaf length and width, panicle length, and grain width (Alamin et al., 2017). The *cfl2* mutant described in the current study was different from all other rolled-leaf mutants. No significant difference in other agronomic traits was observed in the *cfl2* mutant (Supplementary Table S1). Leaves of the *cfl2* mutant were normal during the entire growth period, except for the curled flag leaf base, and the distal portion of the flag leaf blade was normally expanded (Figures 1E,F). However, in addition to the specifically curled flag leaf, the dwarfism phenotypes were also observed in the two allelic mutants *cfl2-1* and *cfl2-2* (Supplementary Figure S2). The *cfl2-1* mutation site located closed to the SRS1 domain, and *cfl2-2* mutation site located in the I-helix domain, both of them showed to be more conserved than that of *cfl2* (Figure 5B), which may explain the different phenotypes in plant height of the three allelic mutants. The unique phenotype of the curled flag leaf in *cfl2*, without defects in plant height or other agronomic traits, provides useful material for the study of the development mechanism of flag leaf.

### Function of *CFL2* in Leaf Development

As the outermost cell layer covering the plant body, the epidermis plays an important role as a protective barrier against biotic or abiotic agents and is an active interface that controls the vital exchange of gas, water, and nutrients with the environment (Javelle et al., 2011). To accommodate its multiple roles, the epidermis has developed a suite of characteristics, including cell types in L1, pavement cells, stomatal guard cells, trichomes, root hairs, papillate cells, and gland cells (Glover, 2000; Martin and Glover, 2007; Javelle et al., 2011). Some epidermis-defective mutants show abnormal leaf morphology, such as the *cfl1* (Wu et al., 2011) and *cld1* (Li et al., 2017) in rice, the *cr4* in maize (Becraft et al., 1996), and the *crinkly4* in *Arabidopsis* (Watanabe et al., 2004). These studies demonstrate that, as a dermal tissue system, the structural integrity of the epidermis is essential to maintain the normal development of leaves. The present SEM observations revealed an increased number and disordered distribution of papillae and an obvious crack on the epidermal of bulliform cell pairs and the linear cork–silica cell pairs in the flag leaf of the *cfl2* mutant (Figures 3A–D,I,J). Other epidermal structures, such as the stomatal and crystal structure of cuticular wax, showed normal development compared with those of the WT (Figures 3E–H,K,L), which indicates that *CFL2* affected the epidermal development only to a particular extent.

As the largest supergene families in plants, cytochrome P450 monooxygenases (CYP450s) play critical roles in the synthesis of lignin, ultraviolet protectants, pigments, defense compounds, fatty acids, hormones, and signaling molecules (Schuler and Werck-Reichhart, 2003). Phylogenetic analysis showed that *CFL2* encoded a cytochrome P450 protein (OsCYP96B4) (Figure 5A), which belongs to the youngest family in CYP86 clan (Nelson et al., 2004). This family is an invention of angiosperms (Nelson and Werck-Reichhart, 2011), implying that the CYP96 family plays important roles in epidermal development. *LCR*, encoding cytochrome P450 CYP86A8, could be implicated in epidermis development and in preventing postgenital organ fusions (Wellesen et al., 2001); *CYP96A15* involved in surface protection by participating in the synthesis of stem epidermis wax in *Arabidopsis* (Greer et al., 2007); *CYP96B5* is involved in the formation of epidermal wax crystals affecting drought sensitivity in rice leaf (Zhang et al., 2020). Several mutants of *OsCYP96B4* have been reported, which point the multi-functionality of *CFL2*/*OsCYP96B4*, in terms of an association with cell elongation and pollen germination (Rengasamy et al., 2011), a role in the fine-tuning of plant height (Zhang et al., 2014), mediation of growth and stress responses by fine-tuning the gibberellin-to-abscisic acid balance (Tamiru et al., 2015), a function in secondary cell wall formation (Wang et al., 2016), and an influence on a variety of metabolic pathways (Jiang et al., 2020). The positive transgenic plants with overexpression of *CFL2* displayed developmental disorders including dwarf and growth retardation and were lethal ultimately (Supplementary Figure S3), which was consistent with previous results (Rengasamy et al., 2011). The dwarfism phenotypes of the two allelic mutants *cfl2-1* and *cfl2-2* also indicated that the *CFL2*/*OsCYP96B4* play an important role in the plant height development (Supplementary Figures S2C,D). In contrast to the dwarfing or brittle sheath phenotypes reported in these allelic mutants of *OsCYP96B4*, the *cfl2* mutant exhibits the specific curled phenotype at the base of the flag leaves without a significant change in plant height and other main agronomic traits (Figure 1, Supplementary Figure S1 and Supplementary Table S1). Besides, the allelic mutant *cfl2-1* and *cfl2-2* also showed the curly flag leaves accompanied by normal morphology of other leaves (Supplementary Figures S2E–H). Results of the GO enrichment analysis of the DEGs and the qRT-PCR showed that genes associated with cellulose biosynthesis and cell wall-related processes were significantly upregulated in the *cfl2* mutant (Figures 8A–C), and the components of cell wall were altered dramatically in the *cfl2* mutant compared with WT (Figure 8D), which suggest the importance of *CFL2* in the cell wall-related processes in the flag leaf and further confirm the versatility of *CFL2*/*OsCYP96B4*. In addition, a previous report indicated that *BSHT1*/*OsCYP96B4* was involved in the expression of the cell wall biosynthesis-related genes in rice plant, especially in sheath (Wang et al., 2016). Consistent with this research, the flag leaf sheath also showed the abnormal twist in the *cfl2* mutant. The highest expression of *CFL2* in the flag leaf sheath may also affect the cell wall-related processes in flag leaf sheath (Figure 6B), which is the possible reason for this phenotype.

### The Relationship Between *Roc5* and *CFL2*

In higher plants, L1 is the outermost cell layer of the SAM and differentiates into all above-ground organs, such as the leaves, stems, and flowers, which are essential for the normal development of the entire shoot (Javelle et al., 2011; Fang et al., 2015). Some genes specially expressed in L1 have been identified, such as *ATML1*, *PDF2*, *ALE2*, and *ACR4* in *Arabidopsis* (Lu et al., 1996; Abe et al., 1999; Watanabe et al., 2004; Tanaka et al., 2007); *ZmOCL1*, *ZmOCL3*, *ZmOCL4*, and *ZmOCL5* in maize (Ingram et al., 1999, 2000); and *ONI1*, *ONI2*, and *ONI3* in rice (Ito et al., 2011; Tsuda et al., 2013; Akiba et al., 2014). The transcription factors of the HD-ZIP IV family are also associated with epidermis development in rice. Nine genes (*Roc1*–*Roc9*) in the HD-ZIP IV family have been identified in rice, of which five are specifically expressed in the epidermis (Ito et al., 2003). Interestingly, mutant of *Roc5* showed similar phenotypes (no significant effect on plant height and enlarged bulliform cells) to the *cfl2* mutant (Zou et al., 2011). The expression levels of *Roc5* and *CFL2* were significantly decreased in the *clf1*, an allelic mutant of *Roc5*, compared with those of “Nipponbare” (Figure 7B). The expression level of *CFL2* was decreased, but the expression of *Roc5* showed no significant difference in the *cfl2* mutant when compared to the WT (Supplementary Figure S4A). Besides, both *Roc5* and *CFL2* showed specific expression in the L1 cell layer of SAM, leaf, and leaf vein (Figures 6D–G), and *Roc5* could bind to the L1 box in the promoter of *CFL2* (Figures 7C–F). These results indicated that transcription factor *Roc5* directly regulated the expression of *CFL2*. The leaves of the *oul1* mutant gradually curved in seedling stage and the curling phenotype became more evident during the growth period (Zou et al., 2011), whereas only the base of flag leaves was affected in the reproductive stage in the *cfl2* mutant (Figures 1E,F). This apparent difference may reflect that, in addition to the regulation of *Roc5*, *CFL2* is also affected by other proteins with currently unknown functions during leaf development, which will be our focus in future studies.

In summary, in contrast to other rolled-leaf mutants, *cfl2* showed specific curling at the base of the flag leaf, whereas the distal portion of the flag leaf blade was normal and the other leaves of the *cfl2* mutant maintained normal development throughout the growth period. Microscopic observations revealed that abnormal epidermis development caused the mutated curled leaf, as a result of enlarged bulliform cells and increased number of papillae with a disordered distribution in the *cfl2* mutant. Biochemical experiments and transcriptome analysis indicate that *CFL2* was controlled by *Roc5*, which bound to the L1 box in the promoter of *CFL2*, and affected epidermis development by influencing the expression of genes associated with cell wall-related processes, thereby regulating leaf morphology (Supplementary Figure S5).

## Data Availability Statement

RNA sequencing data were deposited in the NCBI Sequence Read Archive (SRA) under accession PRJNA628038.

## Author Contributions

XS, GH, and XZha planned and designed the research. YW, XZhu, and XW analyzed the data. XZha, YW, ZZ, YX, and JX performed the experiments. XZha, YW, ZZ, ZY, and XS conducted the fieldwork. XZha, YW, XZhu, XW, XS, and GH wrote, reviewed, and edited the manuscript. All authors contributed to the article and approved the submitted version.

## Conflict of Interest

The authors declare that the research was conducted in the absence of any commercial or financial relationships that could be construed as a potential conflict of interest.

## Footnotes

1 http://rgp.dna.affrc.go.jp

2 http://www.ncbi.nlm.nih.gov

3 http://www.arabidopsis.org

4 https://phytozome.jgi.doe.gov

5 http://bioinfo.cau.edu.cn/agriGO/

6 http://www.gramene.org/

## Supplementary Material

Supplementary Figure 1 Internode comparison of WT and *cfl2*. **(A,B)** Panicle and internode length of WT and *cfl2*. Scale bars = 8 cm. **(C)** Statistical data of internodes in WT and *cfl2.* Mean of five independent experiments. I–VI: the 1st–6th internodes.

Supplementary Figure 2 Phenotype of WT and the allelic mutants of *CFL2*. **(A–D)** Phenotype of WT, *cfl2*, and its two allelic mutants *cfl2*-1 and *cfl2*-2 in tillering stage. Scale bars = 20 cm. **(E–H)** The top three leaves of WT, *cfl2*, *cfl2*-1, and *cfl2*-2. Scale bars = 3 cm.

Supplementary Figure 3 Phenotype of the *CFL2* over-expressed transgenic lines. **(A,B)** Phenotype analysis of the five over-expressed *CFL2* transgenic lines at the seeding stage **(A)** and the mature stage **(B)**. Scale bar = 10 cm **(A)** and 20 cm **(B)**. **(C)** The expression of *CFL2* in the five over-expressed transgenic lines (OE). Values are means ± SD (*n* = 3). Student’s *t*-test was used for statistical analysis (^∗∗∗^*P* < 0.001).

Supplementary Figure 4 Relative gene expression level analysis in wild type and mutants. **(A)** Relative expression level of *Roc5* and *CFL2* in WT and *cfl2.* **(B)** Relative expression level of cell wall-related genes in Nipponbare (Nip) and *clf1*. Values are means ± SD (*n* = 3). Student’s *t*-test was used for statistical analysis (^∗∗^*P* < 0.01).

Supplementary Figure 5 A model of *CFL2*, controlled by *Roc5*, influences flag leaf development in rice by affecting cell wall-related process.

Supplementary Table 1 Agronomic traits of WT and *cfl2*. Mean ± SD of seven biological repeats. Student’s *t*-test was used for statistical analysis.

Supplementary Table 2 The list of annotated genes within the mapping interval.

Supplementary Table 3 Summary of RNA sequencing data.

Supplementary Table 4 Primers used in this study.

Supplementary Table 5 Statistical results of bulliform cell number and areas.

Supplementary Table 6 Original data of the cell wall components analysis.
