# Supplementary figures and images for: Curled Flag Leaf 2, Encoding a Cytochrome P450 Protein, Regulated by the Transcription Factor Roc5, Influences Flag Leaf Development in Rice
Source: Front Plant Sci. 2021 Feb 12;11:616977. doi: 10.3389/fpls.2020.616977 (PMC7907467; doi:10.3389/fpls.2020.616977)

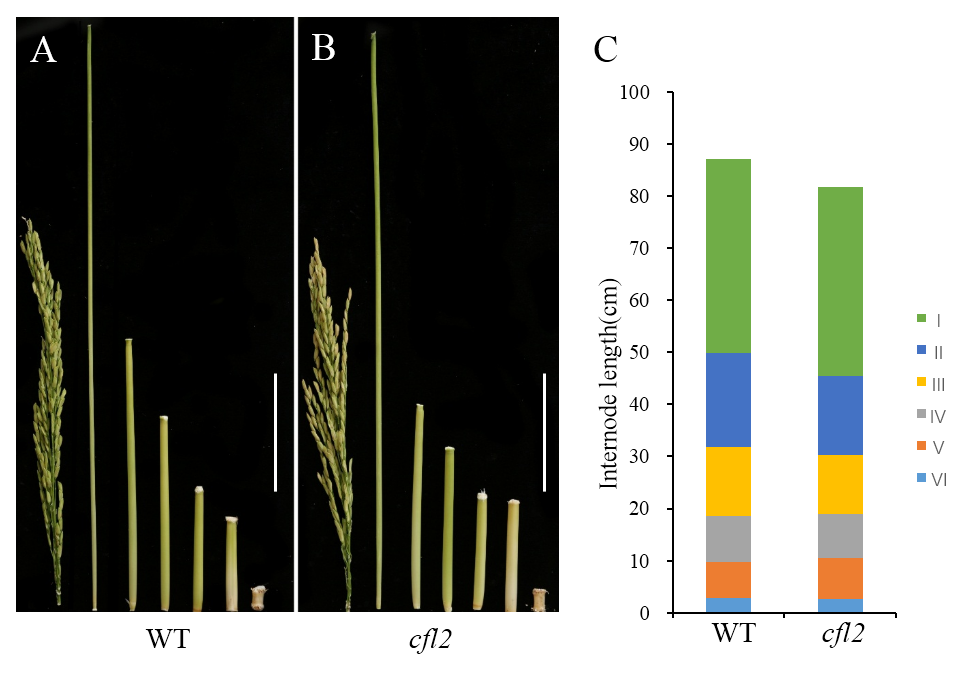

Supplement: Supplementary Figure 1 — Internode comparison of WT and cfl2. (A,B) Panicle and internode length of WT and cfl2. Scale bars = 8 cm. (C) Statistical data of internodes in WT and cfl2. Mean of five independent experiments. I–VI: the 1st–6th internodes. [file Image_1.TIF]

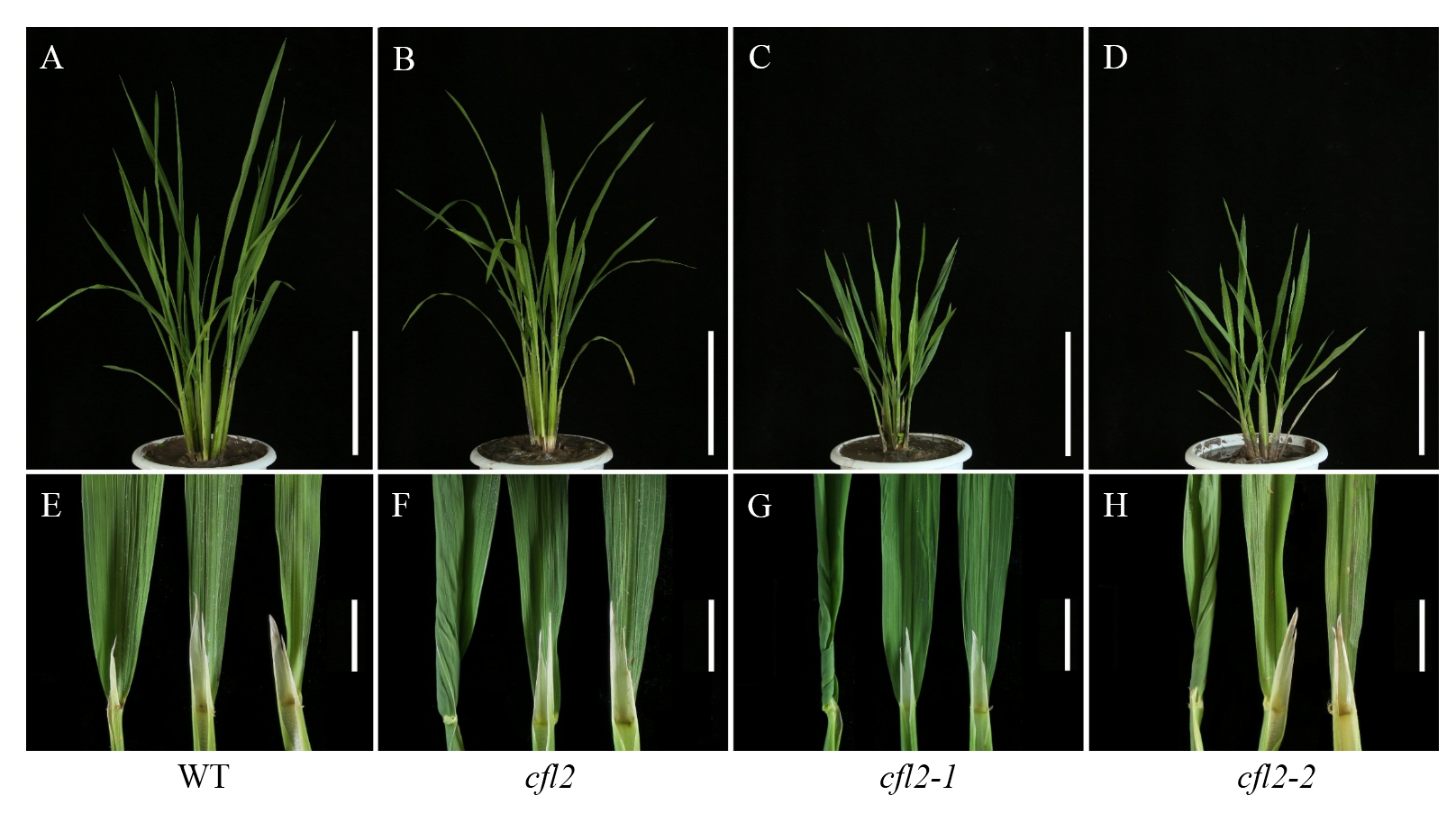

Supplement: Supplementary Figure 2 — Phenotype of WT and the allelic mutants of CFL2. (A–D) Phenotype of WT, cfl2, and its two allelic mutants cfl2-1 and cfl2-2 in tillering stage. Scale bars = 20 cm. (E–H) The top three leaves of WT, cfl2, cfl2-1, and cfl2-2. Scale bars = 3 cm. [file Image_2.TIF]

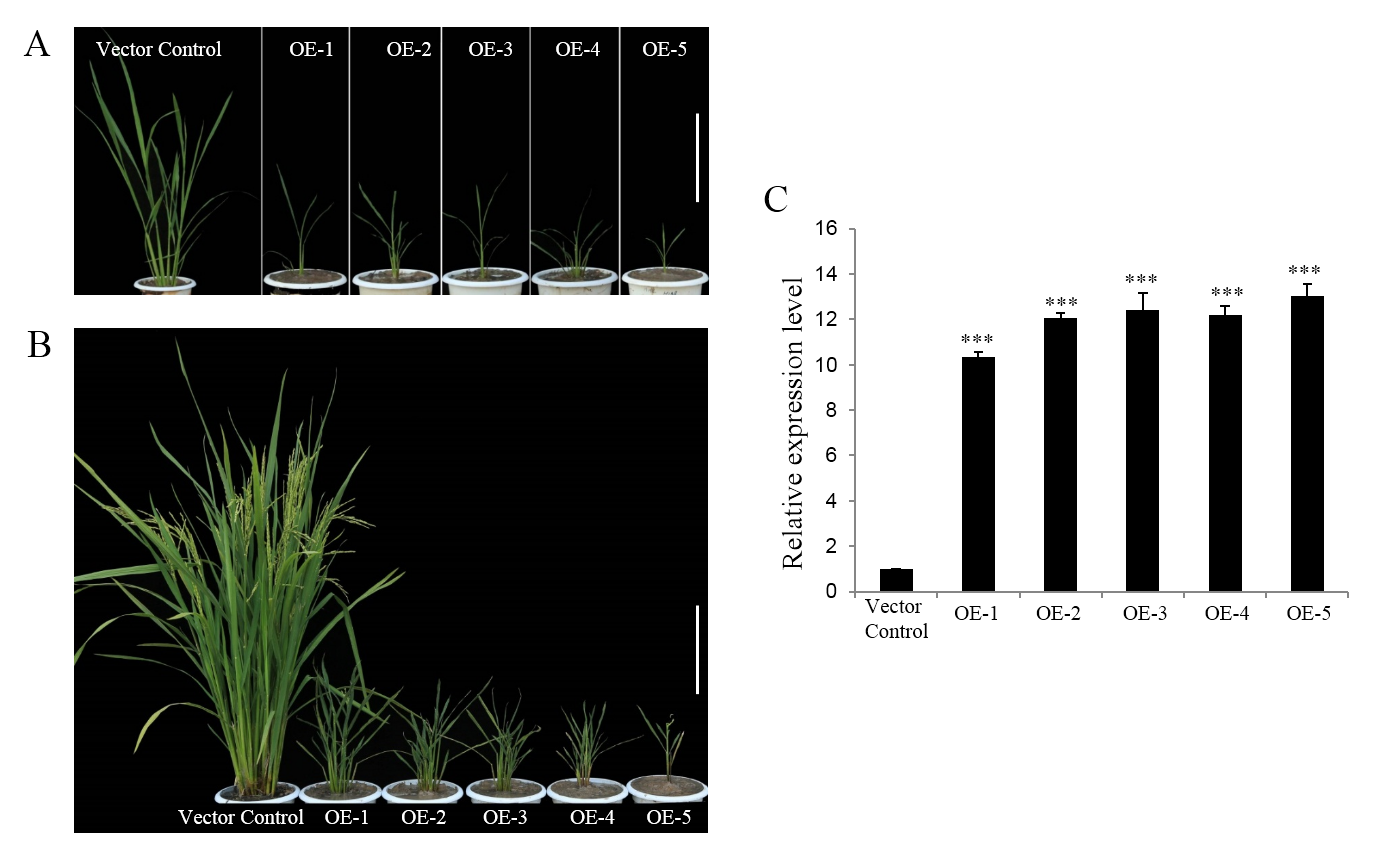

Supplement: Supplementary Figure 3 — Phenotype of the CFL2 over-expressed transgenic lines. (A,B) Phenotype analysis of the five over-expressed CFL2 transgenic lines at the seeding stage (A) and the mature stage (B). Scale bar = 10 cm (A) and 20 cm (B). (C) The expression of CFL2 in the five over-expressed transgenic lines (OE). Values are means ± SD (n = 3). Student’s t-test was used for statistical analysis (∗∗∗P < 0.001). [file Image_3.TIF]

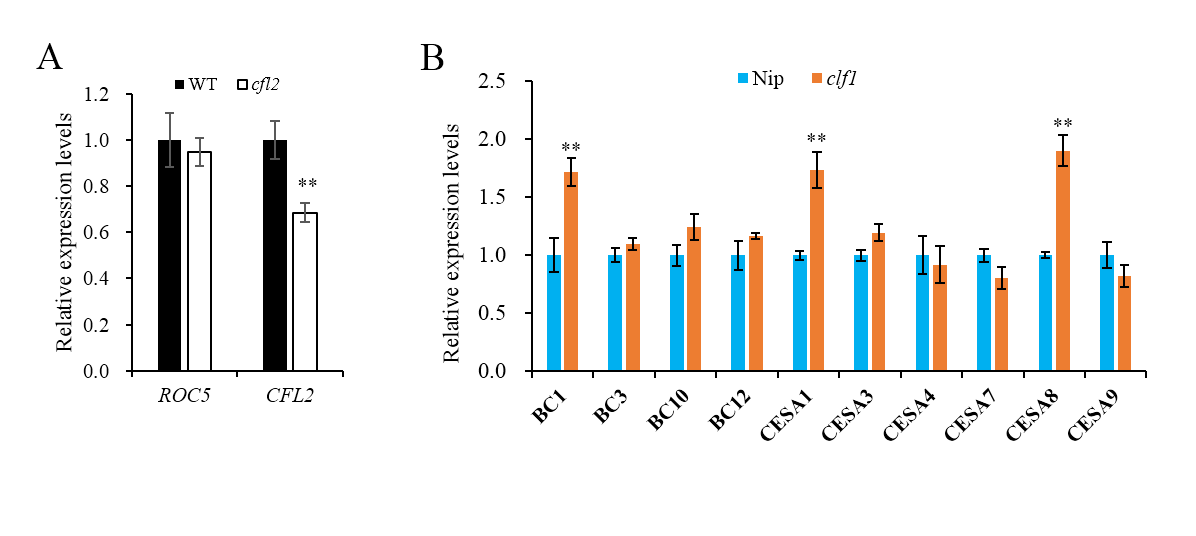

Supplement: Supplementary Figure 4 — Relative gene expression level analysis in wild type and mutants. (A) Relative expression level of Roc5 and CFL2 in WT and cfl2. (B) Relative expression level of cell wall-related genes in Nipponbare (Nip) and clf1. Values are means ± SD (n = 3). Student’s t-test was used for statistical analysis (∗∗P < 0.01). [file Image_4.TIF]

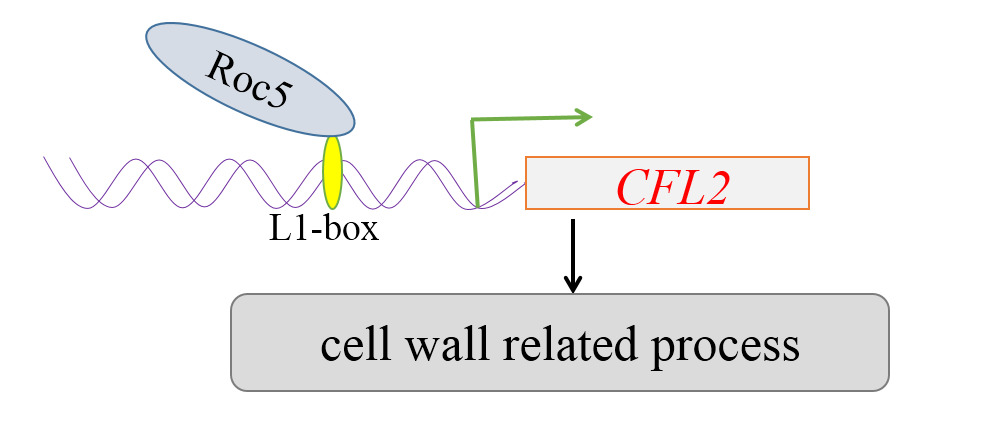

Supplement: Supplementary Figure 5 — A model of CFL2, controlled by Roc5, influences flag leaf development in rice by affecting cell wall-related process. [file Image_5.TIF]

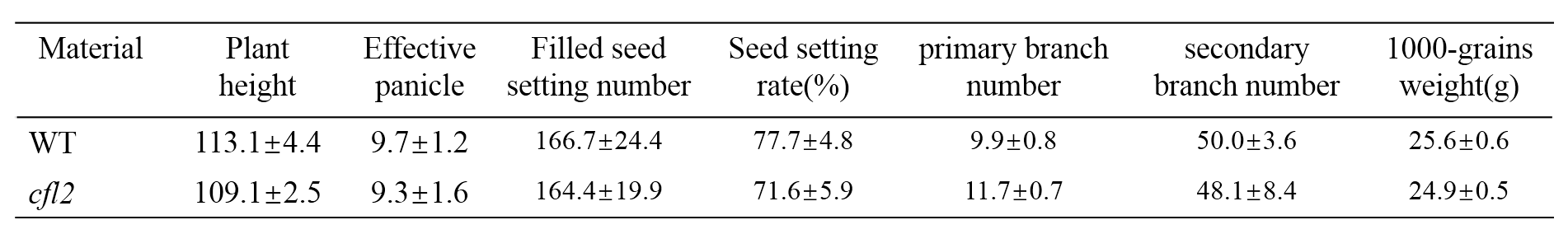

Supplement: Supplementary Table 1 — Agronomic traits of WT and cfl2. Mean ± SD of seven biological repeats. Student’s t-test was used for statistical analysis. [file Image_6.TIF]

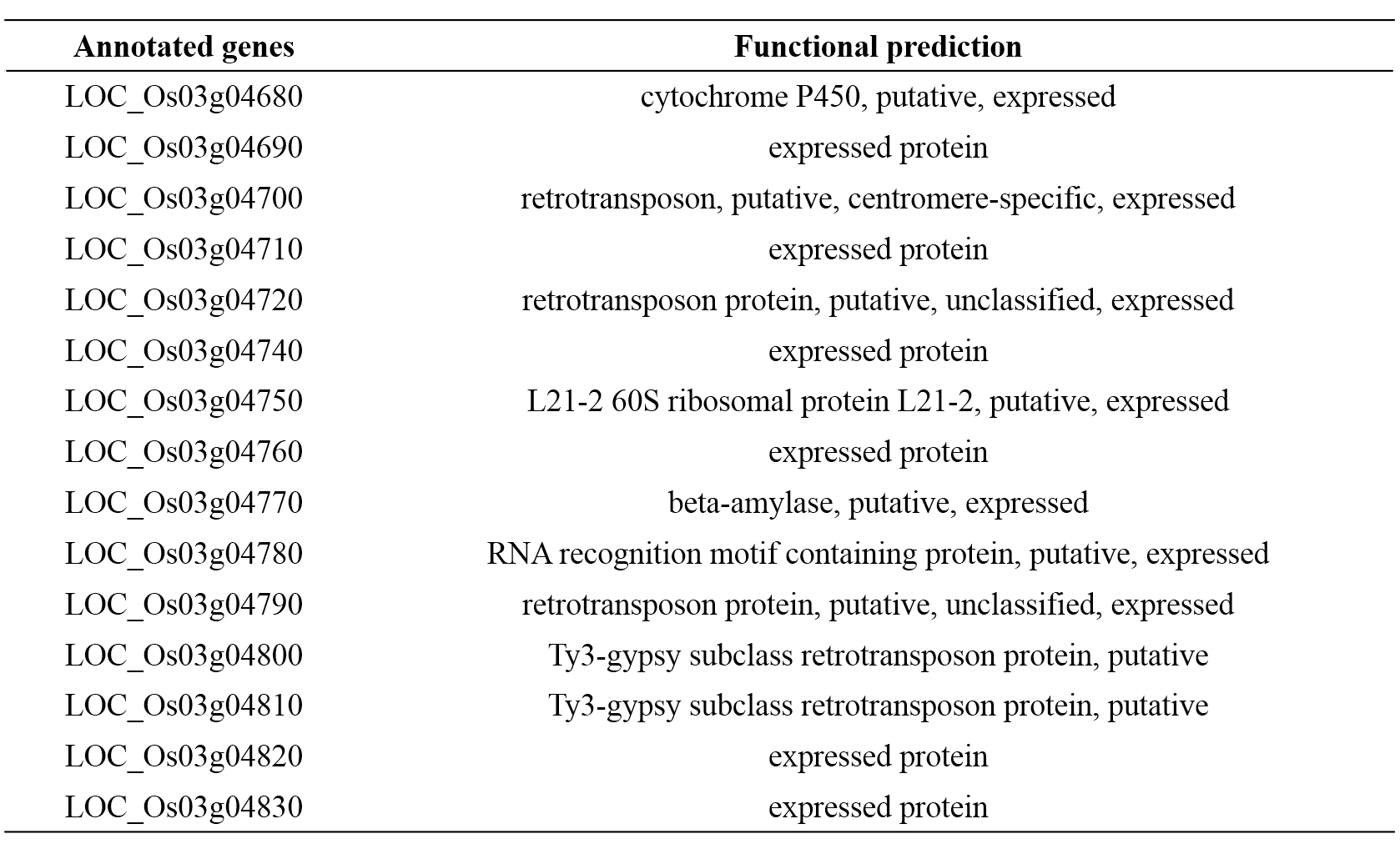

Supplement: Supplementary Table 2 — The list of annotated genes within the mapping interval. [file Image_7.TIF]

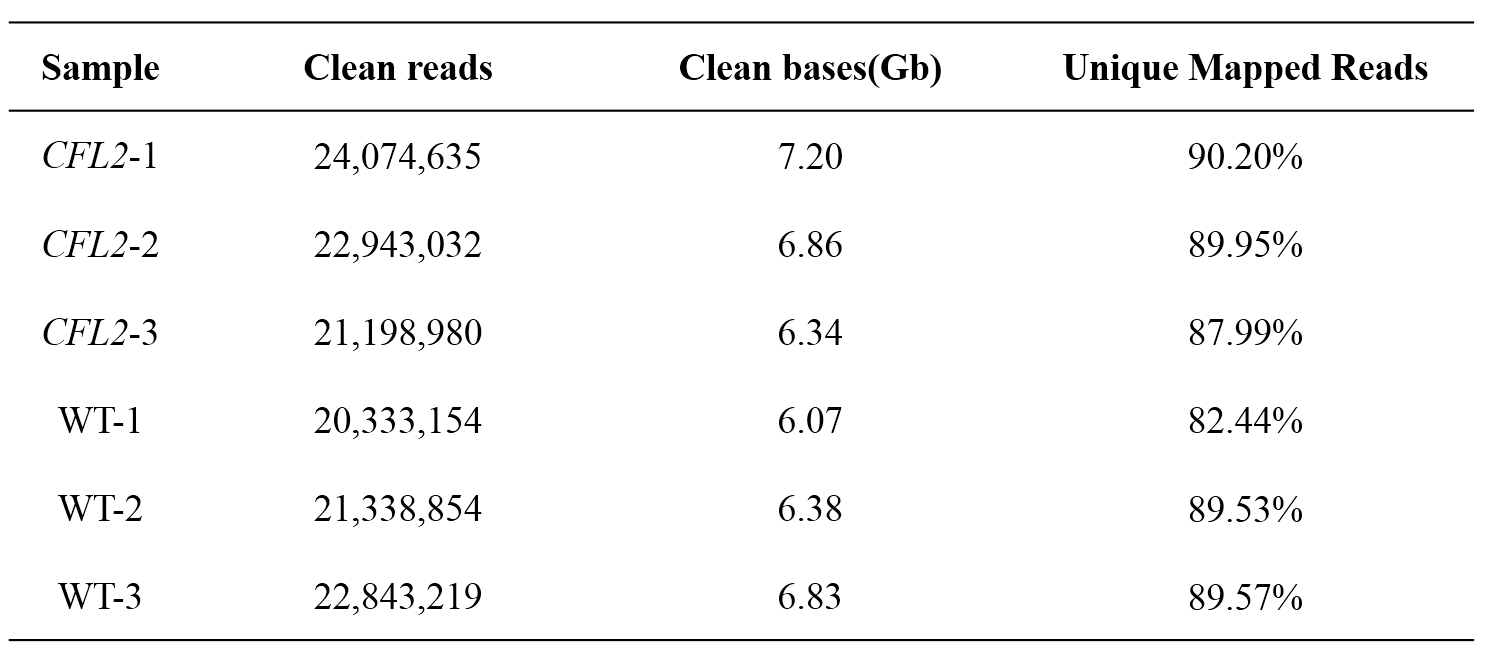

Supplement: Supplementary Table 3 — Summary of RNA sequencing data. [file Image_8.TIF]
